# A large family with MSH3-related polyposis

**DOI:** 10.1007/s10689-022-00297-x

**Published:** 2022-06-08

**Authors:** Arthur S. Aelvoet, Daniël R. Hoekman, Bert J. W. Redeker, Jitske Weegenaar, Evelien Dekker, Carel J. M. van Noesel, Floor A. M. Duijkers

**Affiliations:** 1 Amsterdam UMC location University of Amsterdam, Department of Gastroenterology and Hepatology, Amsterdam, the Netherlands; 2 Amsterdam UMC location University of Amsterdam, Department of Human Genetics, Meibergdreef 9, 1105 AZ Amsterdam, the Netherlands; 3 Amsterdam UMC location University of Amsterdam, Department of Pathology, Amsterdam, the Netherlands; 4 Cancer Center Amsterdam, Amsterdam, the Netherlands; 5 Amsterdam Gastroenterology Endocrinology Metabolism, Amsterdam, the Netherlands

**Keywords:** Adenomatous polyposis, Colorectal cancer, MSH3, MSI, EMAST

## Abstract

Biallelic *MSH3* germline variants are a rare cause of adenomatous polyposis as yet reported in two small families only. We describe the phenotype of a third family, the largest thus far, with adenomatous polyposis related to compound heterozygous *MSH3* pathogenic variants. The index patient was a 55-years old male diagnosed with rectal cancer and adenomatous polyposis (cumulatively 52 polyps), with a family history of colorectal polyposis with unknown cause. Next-generation sequencing and copy number variation analysis of a panel of genes associated with colorectal cancer and polyposis revealed compound heterozygous germline pathogenic variants in the *MSH3* gene. Nine out of 11 siblings were genotyped. Three siblings carried the same compound heterozygous *MSH3* variants. Colonoscopy screening showed predominantly right-sided adenomatous polyposis in all compound heterozygous siblings, with a cumulative number of adenomas ranging from 18 to 54 in an average of four colonoscopies, and age at first adenoma detection ranging from 46 to 59. Microsatellite analysis demonstrated alterations at selected tetranucleotide repeats (EMAST) in DNA retrieved from the rectal adenocarcinoma, colorectal adenomas as well as of normal colonic mucosa. Gastro-duodenoscopy did not reveal adenomas in any of the four patients. Extra-intestinal findings included a ductal adenocarcinoma in ectopic breast tissue in one female sibling at the age of 46, and liver cysts in three affected siblings. None of the three heterozygous or wild type siblings who previously underwent colonoscopy had adenomatous polyposis. We conclude that biallelic variants in *MSH3* are a rare cause of attenuated adenomatous polyposis with an onset in middle age.

**Supplementary Information:**

The online version contains supplementary material available at 10.1007/s10689-022-00297-x.

## Background

In most polyposis patients with hundreds to thousands of colorectal adenomas, pathogenic germline variants in the *APC* or *MUTYH* gene are detected. However, genetic testing of patients with attenuated adenomatous polyposis coli, defined as having 10–100 adenomas, often does not reveal pathogenic variants in known predisposing genes. In 15% of patients with attenuated adenomatous polyposis, a pathogenic germline variant in the *APC* gene is found, and in 35% biallelic variants in the *MUTYH* gene [1–3]. More recently, *APC* mosaicism was found to be a cause of colorectal polyposis and in addition other predisposing genes have been detected such as *POLE*, *POLD1* and *NTHL1* [4, 5]. Pathogenic variants in these genes account for a small number of patients with adenomatous polyposis. Altogether, whereas a genetic cause is suspected in a large proportion of patients with attenuated adenomatous polyposis, in approximately half of them no germline alterations are found.

Pathogenic germline variants in mismatch repair genes, such as *MLH1*, *MSH2*, *MSH6* and *PMS2*, are associated with Lynch syndrome and result in microsatellite instability (MSI) and an accelerated adenoma-carcinoma sequence [6]. Lynch syndrome leads to colorectal cancer but not to adenomatous polyposis. Interestingly, Adam et al. [7] recently identified biallelic pathogenic variants in the mismatch repair gene *MSH3* in four patients with adenomatous polyposis from two unrelated families. Analysis of tumor tissue showed Elevated Microsatellite Alterations at Selected Tetranucleotide repeats (EMAST) instead of MSI of mono- and dinucleotide repeats as typically seen in Lynch syndrome. Next to colorectal adenomas, in these patients colorectal cancer, gastric cancer, astrocytoma, duodenal adenomas, thyroid adenomas and intraductal papillomas were observed. Consequently, *MSH3* was added to the diagnostic panel of genes used in our hospital to screen patients with colorectal polyposis.

Recently, we identified a large family with adenomatous polyposis caused by biallelic *MSH3* pathogenic variants. We describe the phenotype of the four affected family members, aiming to further delineate the phenotype of MSH3-related adenomatous polyposis.

## Case description

**Fig. 1:**
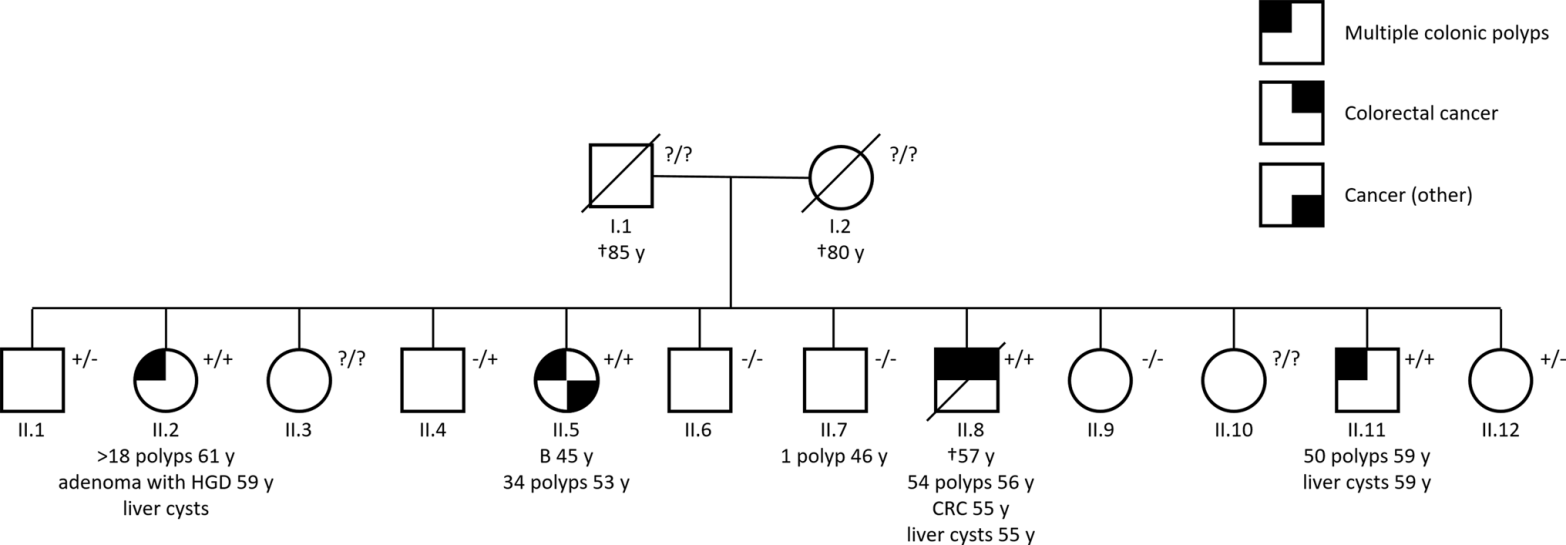
Pedigree of the family. Birth order has been scrambled to protect anonymity. Shown is the cumulative number of polyps and the age at last colonoscopy. Comorbidities are shown with age at diagnosis. Genotype is indicated using +/- (c.2409C > A heterozygous), −/+ (c.(1340 + 1_1341-1)_(2655 + 1_2656-1)del heterozygous), +/+ (compound heterozygous), or ?/? (unknown). *B* breast cancer, *CRC* colorectal cancer, *HGD* high grade dysplasia

A 55-year old patient (Fig. 1, subject II.8) who was recently diagnosed with rectal cancer and colorectal polyposis, having a family history of colorectal polyposis with unknown cause, was referred for genetic testing. Next-generation sequencing (NGS) and NGS-based copy number variation analysis (using a read-depth approach) of a panel of genes associated with colorectal cancer and/or polyposis was performed on DNA isolated from peripheral blood. Compound heterozygous variants c.2409C > A p.(Cys803*) and c.(1340 + 1_1341-1)_(2655 + 1_2656-1)del p.(?), a deletion of exons 9–19, were found in the *MSH3* gene (NM_002439.4) (supplementary material). Both variants are predicted to result in loss of *MSH3* expression due to nonsense-mediated mRNA decay. Both variants were classified as pathogenic (class 5) according to the ACMG guidelines [8]. No other potentially pathogenic variants were found.

Subsequently, all 11 siblings were invited for targeted analysis of the *MSH3* variants c.2409C > A p.(Cys803*) and c.(1340 + 1_1341-1)_(2655 + 1_2656-1)del p.(?), using Sanger sequencing and multiplex ligation-dependent probe amplification (MLPA) with custom made probes for *MSH3*, respectively. Two siblings, both without a history of polyposis or cancer, declined. Two siblings with a known history of adenomatous polyposis (Fig. 1, subjects II.2 and II.5) carried the same compound heterozygous *MSH3* variants, as well as one other sibling (II.11) without a known polyposis. All four subjects carrying compound heterozygous variants were invited to undergo colonoscopy and gastro-duodenoscopy. Furthermore, previous medical records including endoscopy reports and histology reports were collected from all participants.

Table 1 summarizes the findings in terms of polyps and colorectal carcinoma detection as well as extra-intestinal lesions in the four subjects with compound heterozygous variants in *MSH3*.These subjects underwent their first colonoscopy at age 46 (> 12 polyps detected), 53 (one polyp detected), 55 (11 polyps detected) and 59 (6 polyps detected). A total of 17 colonoscopies were performed in these subjects revealing colorectal adenomatous polyposis in all four. A cumulative number of 18, 34, 50 and 54 polyps were detected and the age at last endoscopy was 61, 53, 59 and 56, respectively. The location of the detected polyps is known for 110/156 polyps: 83 (75%) in the proximal colon (cecum, ascending and transverse colon) and 27 (25%) in the distal colon (descending colon and rectosigmoid). Interestingly, the compound heterozygous subject without a known history of colorectal polyposis (II.11) underwent a full colonoscopy at the age of 53, which revealed only one small adenomatous polyp, for which no follow-up examinations were ordered. After genotyping 5 years later at the age of 58, a cumulative number of 49 colorectal polyps were detected during two successive colonoscopies performed within 1 year. In this subject, also an appendicular mucocele was observed without lymphadenopathy on imaging. Appendectomy revealed a tubular adenoma with low-grade dysplasia.

**Table 1:** Three families reported with *MSH3*-related polyposis coli

	Age at diagnosis of polyposis (in years)	Total number of colorectal polyps	Age at last colonoscopy	Total number of gastric polyps	Total number of duodenal polyps	Occurrence of malignancy or high grade dysplastic polyps (age at diagnosis in years)	Benign extra intestinal lesions (age at diagnosis in years)
Family 1 Adam et al. [7] c.1148delA, c.3001 − 2A > C	NR	> 1	NR	NR	> 1	Rectal adenocarcinoma (56) Signet cell gastric carcinoma (59)	Small bilateral renal cysts
	36	≥ 40	NR	NR	> 1		Thyroid adenoma (35) Uterine polyp and leiomyomas (44) Intraductal papillomas mammary glands (44)
Family 2 Adam et al. [7] c.2319 − 1G > A, c.2760delC	32	> 1	NR	NR	> 1	Astrocytoma (26)	Ovarian cysts, including dermoid cyst (27) Uterine myoma (34) Follicular thyroid adenomas (43) Cutaneous fibrolipoma (43) Flat epithelial atypia, multiple peripheral small intraductal papillomas, usual ductal hyperplasias, and cysts with apocrine metaplasia in mammary glands (46)
	33	> 1	NR	NR	NR		
Family 3 (present study) c.2409C > A, c.(1340 + 1_1341-1)_(2655 + 1_2656-1)del	46	34	53	0	0	Primary carcinoma of ectopic axillary breast tissue (45), colorectal adenoma with high-grade dysplasia (46)	
	55	54	56	0	0	Rectal adenocarcinoma (55)	Liver cysts (55)
	53	50	59	0	0		Liver cysts (59)
	59	> 18	61	0	0	Colorectal adenoma with high-grade dysplasia (59)	Liver cysts

*NR* not reported

Among the 146 resected polyps originating from the four subjects with compound heterozygous *MSH3* variants, 138 (95%) were histologically classified as tubular adenomas, 4 (3%) sessile serrated lesions, 2 (1%) traditional serrated adenomas and 2 (1%) as hyperplastic polyps respectively. High grade dysplasia was found in 3 (2%) of the 138 adenomas.

Gastroduodenoscopy in all four affected siblings did not reveal any polyps. Subject II.8 had Barrett’s esophagus with reflux esophagitis and low-grade dysplasia.

With respect to extra-intestinal findings, one compound heterozygous subject (II.5) was diagnosed with adenocarcinoma in ectopic axillary breast tissue at the age of 46, and three (II.2, II.8 and II.11) of the four had liver cysts.

Three of the six siblings with heterozygous or wild type *MSH3* variants previously underwent one or more colonoscopies. Age at last colonoscopy ranged from 50 to 64. Two of them had no colorectal polyposis. In 1 heterozygous sibling, a single adenoma was detected at the age of 46. Follow-up colonoscopy in this patient at age 50 showed no polyps. All participating siblings aged ≥ 55 without the compound heterozygous *MSH3* variants participated in the Dutch national colorectal cancer screening program (biannual faecal immunochemical testing (FIT) between the ages of 55 and 75), and have all received negative FIT-results so far.

**Fig. 2:**
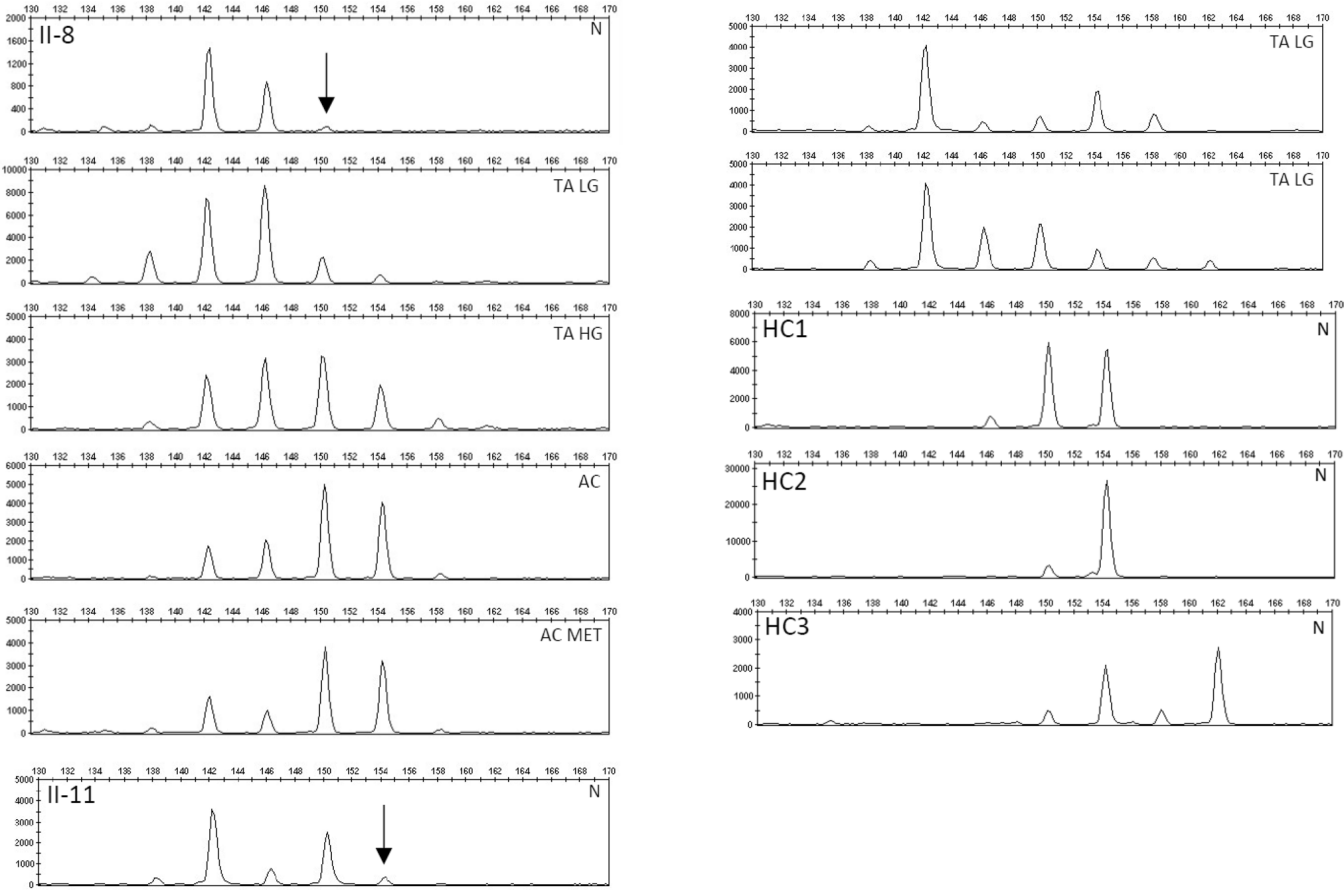
EMAST analyses using the tetranucleotide the marker vWA performed on normal colonic mucosa, tubular adenomas and (metastatic) adenocarcinoma derived from indexpatient II.8 and sibling II.11 and on normal tissue of three healthy donors. Arrows highlight tetranucleotide repeat shifts in present in normal tissue DNA of II.8 and II.11 and not present in normal DNA of three healthy control individuals (HC1–HC3). *N* normal mucosa, *TA* tubular adenoma, *LG* low grade, *HG* high grade, *AC* adenocarcinoma, *MET* metastasis

Immunohistochemical evaluation of the rectal adenocarcinoma of II.8 revealed expression of the mismatch repair genes *MLH1*, *MSH2*, *MSH6* and *PMS2*. Conventional microsatellite analyses using the international standard markers (NR-21, NR-24, MONO-27, BAT25, and BAT26) demonstrated no MSI, but instead did show EMAST (Fig. 2). EMAST was also observed in the adenomas of subjects II.8 and II.11 respectively. Of note, discrete but convincing EMAST was also detected in DNA isolated from normal mucosa of both II.8 and II.11. By next-generation sequencing targeted on a panel of genes associated with colorectal cancer we found two nonsense mutations in the APC gene [c.3467_3470del, p.(Glu1156Glyfs*8) and c.3921_3925del, p.(Glu1309Aspfs*4)] in respectively 38% and 22% of the reads, as well as a two missense mutations in the KRAS gene [c.34G > T, p.(Gly12Cys) and c.40G > A, p.(Val14Ile)] in respectively 35% and 36% of the reads. A more detailed description of material and methods are provided as supplementary material.

## Discussion

We describe the third and largest family reported thus far with *MSH3*-related adenomatous polyposis. Table 1 summarizes the phenotype of all patients reported in literature, including our study. In the current family, the age at diagnosis of polyposis was in the 40’s and 50’s whereas this was in the 30’s in the families reported by Adam et al. [7] Of note, in sibling II.11, only one adenoma was detected at colonoscopy at age 53, but subsequently adenomatous polyposis (cumulatively 50 polyps) was detected at age 59. This late onset of polyposis highlights the importance of pre-symptomatic genetic testing for rare polyp syndromes in all family members despite a negative colonoscopy earlier. The age of onset and severity of polyposis may, next to the biallelic *MSH3* variants, be influenced by differences in other genetic and/or environmental factors. The polyps in the four subjects in the present study were primarily located in the proximal colon (75%), as also seen in attenuated familial adenomatous polyposis and *MUTYH*-associated polyposis. Moreover, in patients with Lynch syndrome with germline mutations in other mismatch repair genes, most cancers are found in the proximal colon. None of the four patients suffered from duodenal adenomas whereas three of the four previously reported patients had duodenal adenomas. Three of four affected subjects had multiple liver cysts. Adam et al. [7] described one affected subject with bilateral renal cysts and a subject with ovarian cysts. As yet, to our knowledge, there is no evidence reported in literature that pathogenic variants of *MSH3* or EMAST are associated with formation of cysts.

Considering the results of the microsatellite analyses (Fig. 2), we conclude that the degree of EMAST does not seem to correlate with the degree of dysplasia, i.e. from adenomas with low grade or high grade dysplasia to cancer, respectively. Interestingly, EMAST was to a low but convincing degree detected in DNA retrieved from normal colon mucosa of the affected subjects (II.8 and II.11), in contrast to DNA isolated from normal embryonic tissues of 10 randomly selected healthy individuals. This suggests diminished DNA repair in somatic (non-tumor) cells of these compound heterozygous individuals. This seems in analogy with the low degree of MSI observed in normal tissue versus high degree of MSI in cancer tissue in patient with bi-allelic Lynch, also called Constitutional MisMatch Repair Deficiency (CMMRD) [9].

Similarly to Lynch syndrome, the adenoma-carcinoma sequence may be accelerated in *MSH3*-related adenomas. Only 4 months after a previous complete colonoscopy (with 21 polyps detected of which four were removed), one of the siblings (II.11) was diagnosed with 40 colorectal polyps. This suggests that new polyps had developed or grown out in a short period of time. We cannot fully exclude that this observation may also be due to inter-observer variability as both endoscopies were performed by different endoscopists. With regard to the carcinogenesis, it is noteworthy that the colorectal carcinoma of the index patient carried pathogenic *APC* and *KRAS* mutations as observed in conventional sporadic and FAP- and MAP-related colorectal carcinomas. Also in a recent study, whole exome sequencing in 9 *MSH3*-related adenomas showed pathogenic somatic *APC* variants in all but one adenoma [10]. Since the index patient had not undergone previous colonoscopies, we are unable to judge whether this would be compatible with an accelerated adenoma-carcinoma sequence.

Endoscopic surveillance in these patients with biallelic pathogenic *MSH3* variants seems warranted. Based on the available data we suggest colonoscopy surveillance every 1 to 2 year from the age of 18, and gastroduodenoscopy surveillance at least every 5 years from the age of 35, with intervals depending on severity of polyposis. Although reported data suggests that endoscopic surveillance potentially could start from a later age, current evidence is too limited and therefore we are careful with providing recommendations. By gaining insight in the natural course of the disease over time, we expect to be able to provide more clear recommendations in the future, also with respect to potential screening for extra-intestinal manifestations for which the data now is too scarce.

## Supplementary Information

Below is the link to the electronic supplementary material.

Supplementary file1 (DOCX 76 kb)

## Data Availability

The data that support the findings of this study are available from the corresponding author (F.D.) upon reasonable request.
