# Supplementary material for: A large family with MSH3-related polyposis
Source: Fam Cancer. 2022 Jun 8;22(1):49–54. doi: 10.1007/s10689-022-00297-x (PMC9829574; doi:10.1007/s10689-022-00297-x)

**Supplementary Material**

**Material and methods**

**Microdissection and DNA extraction**

Cells of interest were microdissected manually out of deparaffinized hematoxylin-stained FFPE tissue sections. DNA was isolated from dissected FFPE tissue by overnight digestion with Proteinase K (1mg/ml; recombinant PCR Grade, 25 mg, Roche) in lysis buffer (50 mM Tris-HCl [pH 8.5], 100 mM NaCl, 1 mM EDTA, 0,5% Tween 20, and 0,5% NP40) at 56°C. For all tests described raw Prot. K lysate was used, no additional DNA purification was performed.

**Microsatellite analysis**

Microsatellite analyses were performed using the Microsatellite Analysis System 1.2 (Promega, Madison, USA), comprising the international standard markers NR-21, NR-24, MONO-27, BAT25, and BAT26. In addition, for evaluation of EMAST the PowerPlex 16 System (Promega, Madison, USA) was used, comprising markers Penta E, D18S51, D21S22, THO1, D3S1358, FGA, TPOX, D8S1179, vWA, Amelogenin, Penta D, CSF1PO, D16S539, D7S820, D13S317 and D5S818. The generated fragments were separated and size selected using the ABI 3730 (Thermofisher) and a size standard (ILS600, Promega). Analysis of the fragments was done using the GeneMapper^TM^ software (ThermoFisher). Powerplex analysis (PP16) was performed using the PowerPlex 16 System (Promega, Madison, USA), comprising the markers Penta E, D18S51, D21S22, THO1, D3S1358, FGA, TPOX, D8S1179, vWA, Amelogenin, Penta D, CSF1PO, D16S539, D7S820, D13S317 and D5S818. For both MSI and EMAST analyses the generated fragments were separated and size-selected using the ABI 3730 (Thermofisher) and a size standard (ILS600, Promega). Analysis of the fragments was done using the GeneMapper^TM^ software (Thermofisher).

**Genetic analysis of germline samples**

Germline mutation analysis of the DNA of the index patient was performed using a custom designed in solution capture (SeqCap EZ Choice, Nimblegen) comprising *APC, MUTYH, BMPR1A, SMAD4, STK11, MLH1, MSH2, MSH6, PMS2, PTEN, POLE, POLD1, ACVRL1, AXIN2, ENG, NTHL1, MSH3, MLH3, RNF43, CDH1, TSC1, TSC2, EPCAM, GREM1* and sequenced on a MiSeq (Illumina). Sequences are analysed using Alissa software (Agilent). CNV detection is performed and CNV boundaries are determined by means of Depth of Coverage per exon. Differences in normalised coverage are used to calculate Z-scores. A schematic representation of the CNV analysis of the *MSH3* gene of the index patient is provided in supplementary figure 1.

Analysis of the DNA of the index patient’s siblings targeted for the *MSH3* mutations c.2409C>A and c.(1340+1_1341-1)_(2655+1_2656-1)del was performed by Sanger sequencing and multiplex ligation-dependent probe amplification (MLPA), respectively. The MLPA kit P003-D1 (MRC Holland) was used combined with the following synthetic custom MLPA probes for exons 12 and 17 of the *MSH3* gene:

MSH3E12-LPO:

**GGGTTCCCTAAGGGTTGGA**CCAAAGGAAGTTTGCTGTGGGTTTTAGACCACACTAAA

MSH3E12-RPO (5’ phosphorylated):

ACTTCATTTGGGAGACGGAAGTTAAAGAAGTGGGTGACCCAGC**TCTAGATTGGATCTTGCTGGCAC**

MSH3E17-LPO:

**GGGTTCCCTAAGGGTTGGA**CAGACATCTGAATCAGCTCCGGGAGCAGCTAGTCCT

MSH3E17-RPO (5’ phosphorylated):

TGACTGCAGTGCTGAATGGCTTGATTTTCTAGAGTG**TCTAGATTGGATCTTGCTGGCAC**

(target specific sequences underlined, primer specific sequences bold)

**Next generation sequencing of tumor samples**

DNA mutation analysis was performed by next generation sequencing using a custom-made Ion AmpliSeq^TM^ Colon panel (ThermoFisher Scientific, Waltham, MA, USA) comprising *APC, BMPR2, BRAF, CTNNB1, FBWX7, KRAS, MLH1, MSH2, MSH3, MSH6, NRAS, PIK3CA, PMS2, POLD1, POLE, RNF43, SMAD4, TP53* and *ZNRF3*. Polymorphic SNPs were included in the genomic regions of *APC, MLH1, MSH2, MSH6* and *PMS2* in order to detect allelic imbalances. The Ion AmpliSeq Library Kit 2.0 was used for library construction. Emulsion PCR and template preparation were executed on the Ion Chef Instrument (ThermoFisher). Sequencing was performed on Personal Genome Machine (ThermoFisher) using 318 V2 Chip and the Ion GeneStudio S5 Prime System using Ion 530 Chips. The average sequencing depth was 1,500 reads. Sequencing data were analysed using SeqNext, version 4.2.1 (JSI medical systems GmbH, Ettenheim, Germany). Variants were called with a threshold of 5%.

**Supplementary figure 1. CNV analysis of the *MSH3* gene**


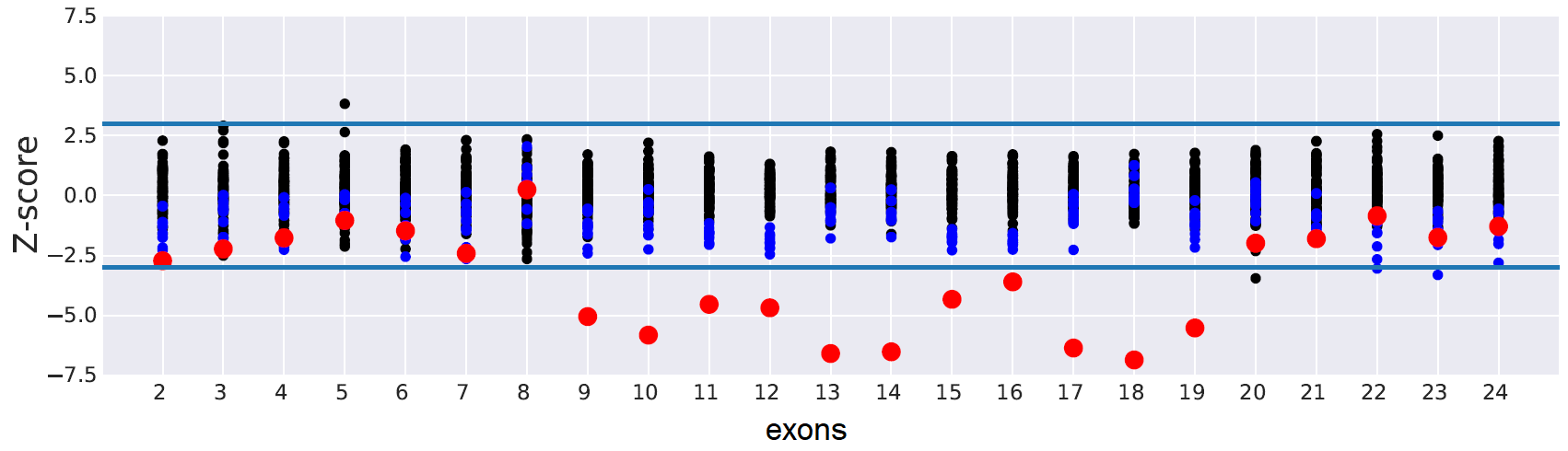

Supplement: Supplementary file 1 — Supplementary file1 (DOCX 76 kb) [file 10689_2022_297_MOESM1_ESM.docx]
